# Successful use of an older ionic contrast media in a patient with systemic hypersensitivity to ioxilan

**DOI:** 10.1186/1939-4551-8-S1-A240

**Published:** 2015-04-08

**Authors:** Smita Joshi, Robert Lin

**Affiliations:** 1 Weill Cornell Medical College, USA

## Background

The use of iodinated contrast media (CM) is commonly associated with hypersensitivity reactions. However, the management of hypersensitivity reactions is not well established especially for delayed hypersensitivity reactions (DHRs). We report a case of CM associated systemic hypersensitivity where selection of a structurally different CM allowed for successful subsequent imaging.

## Methods

Case report.

## Results

A 78-year-old female with end stage renal disease on hemodialysis via arteriovenous fistula, diabetes mellitus, hypertension, and hyperlipidemia was evaluated for recurrent fever and rash after receiving CM for a fistulogram. She had received prednisone prophylactically for reactions associated with ioxilan administration on two prior fistulograms. These reactions consisted of diffuse erythematous rash preceded by fever that started 2-4 hours after contrast administration and lasted several days. After receiving pretreatment with prednisone and diphenhydramine, she received her most recent dose of ioxilan (70 mL) for a fistulogram and developed fever and diffuse erythematous rash (2 and 4 hours post injection, respectively). The fever lasted 3 days and the rash subsequently desquamated involving the arms, trunk, neck and face. Her skin fully re-epithelialized 2 weeks later. She had negative skin prick tests and negative patch tests to ioxilan, iothalamate, and iohexol. She subsequently underwent challenge with 10 mL of intravenous iothalamate and was observed for 4 hours without incident. Later, she underwent fistulogram with iothalamate and tolerated it without adverse reactions.

## Conclusions

Hypersensitivity reactions are generally much less common with non-ionic CM compared to older ionic CM. However, for DHRs, reaction rates between ionic CM and non-ionic CM are similar. Successful administration of different CM selected based on skin tests has been reported for patients with previous DHRs to specific CM. In our case, skin testing was non-diagnostic, and we hypothesized that the distinct chemical structures of these agents would make immunological cross-reactivity unlikely. Indeed this patient who was repeatedly reactive to the non-ionic low-osmolal CM ioxilan, subsequently tolerated the ionic hyperosmolal CM iothalamate. We conclude that different epitopes involved in DHRs to CM may be suggested based on differing chemical structure and ionicity. Further research is needed regarding both the cross-reactivity and cross-tolerance of various CM in DHRs, especially in CM skin-test negative patients.

## Consent

Written informed consent was obtained from the patient for publication of this abstract and any accompanying images. A copy of the written consent is available for review by the Editor of this journal.

